# Feasibility Study of Back2School, a Modular Cognitive Behavioral Intervention for Youth With School Attendance Problems

**DOI:** 10.3389/fpsyg.2020.00586

**Published:** 2020-04-06

**Authors:** Johanne Jeppesen Lomholt, Daniel Bach Johnsen, Wendy K. Silverman, David Heyne, Pia Jeppesen, Mikael Thastum

**Affiliations:** ^1^Department of Psychology and Behavioural Sciences, Aarhus University, Aarhus, Denmark; ^2^TrygFonden’s Centre for Child Research, Aarhus University, Aarhus, Denmark; ^3^Yale University Child Study Center, New Haven, CT, United States; ^4^Institute of Psychology, Leiden University, Leiden, Netherlands; ^5^Child and Adolescent Mental Health Center, Mental Health Services – Capital Region of Denmark, Gentofte, Denmark; ^6^Department of Clinical Medicine, Faculty of Health and Medical Sciences, University of Copenhagen, Copenhagen, Denmark

**Keywords:** Back2School, school attendance problems, cognitive behavioral therapy, transdiagnostic, feasibility, acceptability, youths

## Abstract

There is large heterogeneity among youth with school attendance problems (SAPs). For this reason, protocols for the treatment of SAPs need to be flexible. Back2School (B2S) is a new manual-based, modular transdiagnostic cognitive behavioral intervention to increase school attendance among youth with SAPs. It also aims to increase the self-efficacy of these youth and their parents. B2S includes evidence-based modules addressing youth anxiety, depression, and behavior problems, together with modules focused on parent guidance and school consultation. The current study examined the feasibility of evaluating B2S in an randomized controlled trial and acceptability of the B2S program in a non-randomized trial, including both qualitative and quantitative data, in preparation for a randomized controlled trial of its effectiveness. Youth, parents, and teachers completed questionnaires at baseline, post-intervention, and follow-up. School attendance data were collected from school registers. Twenty-four youth with a SAP (defined as more than 10% absenteeism during the last 3 months) were recruited from primary and lower secondary schools in Aarhus Municipality, Denmark. Their parents also participated in B2S. Two of the 24 families withdrew during the intervention, after sessions two and six respectively. Of the remaining 22 families, 19 (86%) completed all 10 sessions. Parents and youth rated their satisfaction with B2S as high, and high levels of satisfaction were maintained 1 year after the intervention. Teacher satisfaction was lower than that of youth and parents, but the majority found the school’s participation in the intervention helpful. Preliminary evaluation of intervention outcomes showed significant increase in school attendance and decrease in psychological symptoms, as well as a significant increase in self-efficacy for both youth and parents. Based on this feasibility data, adaptations were made to the B2S manual and study procedures prior to commencement of a randomized controlled effectiveness trial. The main adaptation to the manual was to increase school consultation. The main procedural adaptation was to broaden recruitment. Furthermore, it was necessary to increase level of staffing by psychologists because treatment delivery was more time consuming than expected.

## Introduction

The school context is important for youths’ academic development and the development of their social-emotional competencies (Kearney and Graczyk, 2014). School absenteeism has a negative impact on development in these areas (Carroll, 2010; Gottfried, 2014). Long-term school absenteeism increases a youth’s risk of early school dropout, which increases the risk of employment, financial, social, and health issues in adulthood (Attwood and Croll, 2006; Christle et al., 2007; Kearney, 2008b).

In the United States and United Kingdom there has been an increase in the number of students with chronic absenteeism (i.e., more than ten percent; Chang et al., 2018; Department for Education, 2019). The increase in absenteeism is also seen in Danish schools. On average, Danish students in elementary and lower secondary school are absent from school 12 days each school year (six percent of school days), representing an increase since 2014/2015 of one whole day of absenteeism (Undervisningsministeriet, 2018). More specifically, there has been a decrease in lower levels of absenteeism (i.e., 0–2% absenteeism) and an increase in higher levels of absenteeism (i.e., more than 10% absenteeism during a school year) (Undervisningsministeriet, 2018).

School attendance problems (SAP) encompasses different types of problematic school absenteeism. There is large heterogeneity among youths with SAPs, whereby etiology, associated psychopathology, and presentation vary according to the type of SAP (e.g., Kearney, 2008a; Heyne et al., 2019). Customarily, interventions to improve school attendance have focused on one specific type of SAP, such as school refusal alone or truancy alone. Moreover, the effectiveness of these interventions has mainly been examined in small-scale studies or without a randomized controlled design (Maynard et al., 2013, 2015).

A functional approach has been developed to address the heterogeneity associated with SAPs. It involves identifying the motivational function of a youth’s SAP, including two motivational functions referring to negative reinforcement such as avoidance of school-based situations or escape from aversive social and evaluative situations, and two motivational functions referring to positive reinforcement such as pursuit of attention from significant others or outside school (Kearney and Silverman, 1993). The functional approach attempts to covers all youth with problematic absenteeism and are linked to an assessment covering both the form and function of SAPs as well as providing treatment strategies targeting different reasons for SAPs. “When Children Refuse School” comprises interventions for absenteeism based on this functional approach, with four protocols to address the four motivational functions (Kearney and Albano, 2007). The strength of the program is the focus on different functions of SAPs. However, the program does not involve interventions at the school.

An intervention which is relevant for different types of SAPs needs to be flexible, containing intervention components most relevant to those different types. There are several risk factors for SAPs related to contexts of the youth as the family context and school context (Kearney, 2008b). These contexts are therefore relevant to take into account in an intervention for SAPs.

Studies have found significant associations between youth with SAPs related to school refusal and internalizing symptoms and emotional disorders (Bools et al., 1990; Egger et al., 2003). For youth with SAPs classified as truancy an association with externalizing problems has been found including a higher frequency of conduct disorder (Bools et al., 1990; Egger et al., 2003; Vaughn et al., 2013). However, despite the link between school refusal and internalizing behavior, depression-related internalizing behavior is not only linked to youth with school refusal, as a link between truancy and depression has been found as well (Roeser et al., 1998; Egger et al., 2003; Heyne et al., 2019).

We developed the Back2School program (B2S; Thastum and Arendt, 2017) which is a modular transdiagnostic CBT intervention aimed at increasing school attendance and decreasing anxiety, depression, and/or behavior problems among youth with SAPs. B2S has a systemic approach involving both the family and the school in the program, Improvement in youth self-efficacy for school-related situations is also targeted in the B2S program because low self-efficacy appears to be related to SAPs (Heyne et al., 1998; Maric et al., 2013; Mann et al., 2015) and an increase in self-efficacy may have a positive impact on school attendance (Heyne et al., 2015).

### Aim

The objectives of the current study were to examine the feasibility of evaluating B2S in an RCT and acceptability of the B2S program in a non-randomized trial, including both qualitative and quantitative data. The results would be used to inform a subsequent randomized controlled trial (RCT) of the efficacy of the B2S program. A feasibility study provides valuable information about improvements that may need to occur before initiating a larger RCT, thereby improving the quality and integrity of the RCT (Orsmond and Cohn, 2015). The feasibility of evaluating B2S in an RCT was examined with respect to: recruitment capability and the resulting sample characteristics; data gathering procedures, including the suitability of selected outcome measures based on response rate and comprehension level; the acceptability of the intervention and study procedures; and the resources needed to implement the study and intervention. The feasibility study also served as a preliminarily evaluation of the impact of the intervention. In these ways, the current study followed the model for feasibility studies as proposed by Orsmond and Cohn (2015). In their review of methods associated with feasibility studies, they identified five overarching objectives, which we have also adopted, namely the evaluation of: recruitment capability and resulting sample characteristics; data collection procedures and outcome measures; acceptability of the intervention and study procedures; ability to manage and implement the study and its intervention; and initial responses to the intervention.

## Materials and Methods

### Participants

We estimated that 24 families would need to be included in the feasibility study to ensure that all five therapists and 12 co-therapists could gain experience delivering the B2S program with at least two cases. Thus, the current sample consisted of 24 youths with SAPs, and their parents. Inclusion criteria for the participating youths were: (1) enrollment in a public school within Aarhus Municipality; (2) aged 7–16 years and in 0–9th grade (excluding second semester of ninth grade); (3) parent reported more than 10% school absenteeism during the last 3 months of school; (4) the youth and at least one of the parents understood and spoke Danish sufficiently to complete questionnaires and participate in the intervention; (5) commitment from both the youth and at least one parent to participate in assessment and intervention procedures; and (6) written informed consent provided by the holders of the parental rights and responsibilities. Regarding the first criterion, private schools were not included because within Aarhus Municipality private schools are outside the municipality’s jurisdiction, rendering school absenteeism data unavailable. Regarding the second criterion, youth in their second semester of ninth grade were excluded because this is the final semester in Danish public schools, after which Aarhus municipality cannot provide absenteeism data.

### Procedure

The study was conducted in collaboration between Aarhus University and Aarhus Municipality, Denmark. The intervention was managed by the Center for Psychological Treatment for Children and Adolescents (CEBU) at Aarhus University. The feasibility study was conducted in the spring of 2017.

The families were required to make initial contact with CEBU to participate in the study. Prior to the start of the study, the municipality implemented widespread and extensive information campaigns aimed at families and professionals within the municipality. The suitability of each family, with respect to study inclusion criteria, was initially assessed by the first or last author based on a brief e-mail sent by the family. The email described the youth’s problems regarding school attendance, as well as an estimate of the youth’s absenteeism from school during the last 3 months. Families deemed eligible received information about the project verbally (by telephone) and then in written form by mail. All parents signed an informed consent form for participation. Included in the consent was permission for the investigators to contact the school and involve the school in the intervention. The youth and one of the parents completed questionnaires administered at four assessment points (baseline, post-intervention, 3-month follow-up, and 12-month follow-up). It was optional which parent completed the questionnaires, but ultimately it was the mothers who completed the questionnaires at all assessment points. The main teacher for the youth also completed questionnaires at three assessment points (baseline, post-intervention, 3-month follow-up). All questionnaires were administered electronically.

### Intervention

The B2S program (Thastum and Arendt, 2017) is a manualized CBT program developed for this study to increase school attendance among youth with SAPs. It was used together with a modular transdiagnostic CBT manual called MindMyMind (MMM; Jeppesen, 2017). The MMM manual includes modules of evidence-based CBT targeting subclinical or clinical levels of anxiety, depression, behavioral disturbance, and trauma-related problems. The MMM manual served as a supplement to the B2S manual, inasmuch as the B2S manual indicated when relevant modules and materials from the MMM manual should be used. Therefore, when referring to the B2S program and intervention in this study it refers to the B2S manual supplemented by the MMM manual.

As previously described (Thastum et al., 2019), the B2S intervention is based on a descriptive functional analysis obtained by the School Refusal Assessment Scale (SRAS) (Kearney and Silverman, 1993) together with a case formulation approach to planning CBT for attendance problems. According to B2S, SAPs motivated by positive reinforcement require CBT procedures such as parent management, contingency management, and contracting to minimize incentives for school absenteeism and boost incentives for attendance. SAPs motivated by negative reinforcement require CBT procedures such as cognitive restructuring and exposure-based practice to reduce the youth’s anxious or depressive physical sensations and thoughts. In the development of the intervention, we were guided in part by “the @School program” (Heyne et al., 2014) and the “When Children Refuse School program” (Kearney and Albano, 2007). The @school program informed the collaboration with school staff during regular meetings at the school (e.g., preparing the youth for return to school) and how to address parent motivation. The “When Children Refuse School” program informed the flexible use of different modules depending on the youth’s underlying problems, as well as the role of negative and positive reinforcement.

Each family receiving the B2S intervention was treated by one psychologist and one co-therapist. The psychologists were employed as school psychologists in Aarhus Municipality or as clinical psychologists at CEBU. Graduate students in clinical psychology at CEBU functioned as co-therapists. All psychologists and co-therapists participated in a 6-day training course and received weekly face-to-face group case supervision by specialists in clinical child psychology.

Before the intervention, youth and parents participated in a 1.5-h structured assessment interview held by the appointed therapists to get an understanding of the youth’s development, family and social situation, SAPs, and functioning in daily life. The interview also included a brief, semi-structured psychopathological interview with the youth and parents together. This interview was based on a psychopathological interview developed for MMM but included questions about the youth’s SAPs. The youth did not receive a psychiatric diagnosis following the assessment, but based on the information derived from the interview and the questionnaires, a case formulation was developed by the therapists. The structure of the case-formulation was based on the framework by Carr (2006), where factors related to the development and maintenance of the youth’s problem were included in the case-formulation. These factors were related to predisposing factors, maintaining factors, protective factors, and precipitating factors (Carr, 2006). The case-formulation was discussed with a clinical psychologist at CEBU, and a preliminary treatment plan was constructed.

The B2S intervention consisted of ten 1-h sessions with the youth and parents together, except for sessions two and six, which were only with the parents. Additional, the B2S intervention consisted of a 1-h booster session with the youth and parents together which were flexible but recommended to be 1–3 months after the last session. Finally the B2S intervention consisted of four school meetings. At week one and two of the intervention there were two sessions per week to speed up the change process. The following six sessions could optionally be scheduled weekly or biweekly as decided by the therapist and the family together.

An important part of the B2S intervention is the collaboration with the school. In addition to the B2S sessions with the family, there were four meetings with relevant school officials from the youth’s school, the therapists, and the parents. The meetings were held at the youth’s school in the beginning, the middle, and the end of the intervention, as well as shortly after the booster session. Table 1 presents an overview of the intervention.

**TABLE 1:** Overview of the Back2School program.

Session number	Duration (hours)	Participants	Session content
S-0	1.5	T, C, P	Structured assessment interview with the family conducted by the therapists (a clinical psychologist and a clinical psychology graduate student). The family receive handouts regarding psychoeducation and SMART goals as homework for session 1.

Clinical conference	1	T	The therapists are discussing the case formulation, choice of treatment modules, and treatment goals with a clinical psychologist at CEBU

S-1	1	T, C, P	Presenting and discussing the case-formulation with the family. Psychoeducation regarding school absence, and development of SMART goals.

S-2	1	T, P	Parent only session 1. Helping the parents to clarify and solve potential questions/problems regarding school placement, somatic symptoms in child, and parental motivation for change. Planning better routines at home. Working with potential sleep problems.

S-3	1	T, C, P	Planning the date for returning to school, and planning the first day back in school. Creating a gradual exposure plan for returning to school.

S-4	1	T, C, P	Psychoeducation regarding the youth’s primary problem related to school absence (anxiety, depression, or behavioral problems) by including the MMM Modules. Continuing work with the gradual exposure plan for returning to school.

S-5	1	T, C, P	Continuing work with CBT methods regarding the youth’s primary problem related to school absence (e.g., exposure, behavioral activation and/or cognitive restructuring) by including the MMM Modules. Continuing work with the gradual exposure plan for returning to school. Working with boundaries.

S-6	1	T, P	Parent only session 2. Working with parent behavior. Identifying and reducing factors at home that maintain school absence.

S-7	1	T, C, P	Continuing to work toward returning to school. Revising gradual exposure plan. Focusing on how parents can support the youth in exposure exercises, and returning to school. Problem solving

S-8	1	T, C, P	Open session tailored to needs of the youth and parents. Continue working with CBT methods by including the MMM Modules. Open session tailored to needs of the youth and parents. Continue working with CBT methods by including the MMM Modules.
S-9	1	T, C, P	

S-10	1	T, C, P	Concluding the program. Focusing on maintaining and continuing the progress.
Booster	1	T, C, P	Focusing on maintaining and continuing the progress. Problem solving regarding relevant problems. Advise possible further help.

SM-1	1	T, P, S	Presenting and discussing the case formulation with the school. Planning the schools role in the youth’s return to school. Informing the school about the B2S and CBT approach.

SM-2	1	T, S	Following up on the youth’s progress in the school setting. Discussing potential academic difficulties, problems regarding bullying or other problems.

SM-3	1	T, S	Planning how the school can continue to help and support the youth. Discussing relapse prevention.

SM-4	1	T, S	Planning how the school can continue to help and support the youth. Discussing relapse prevention.

*S, session; SM, school meeting; C, child; P, parent; T, therapist; S, school officials. The table is published in Thastum et al. (2019).*

### Feasibility Measures

#### Sample Characteristics

Measures were collected at baseline, post, 3-months follow-up, and 12-months follow-up. At baseline, parents completed questions regarding family demographics, socioeconomic status, and the youths’ and parents’ mental and physical health. At post, 3-months follow-up, and 12-months follow-up, the parents were asked to report if there were changes to their background information. Also at baseline, youth and parents provided a functional assessment of the youth’s SAPs by completing an adapted version of the *School Refusal Assessment Scale-revised* (SRAS-R; Kearney, 2002; Heyne et al., 2017). The SRAS-R includes four subscales each representing a functional condition of school refusal in youths: (1) avoid stimuli that provoke negative affectivity, (2) escape aversive social and/or evaluative situations, (3) pursue attention from significant others, and/or (4) pursue tangible re-enforcers outside of school. The SRAS-R consists of a youth and parent version, both including 24 items rated on a 7-point scale ranging from 0 to 6. The function with the highest combined score from both the youth and parent version is classified as the primary function of the SAPs and are hypothesized to be the primary maintaining variable of the youth’s SAPs. Functional scores within 0.25 points of one another are considered equivalent (Kearney et al., 2004).

#### Evaluation of Data Gathering Feasibility

Response rate for completing the questionnaires for all informants were evaluated at each data collection point.

#### Resources to Implement the Study

The intervention and study procedure were evaluated at post with the psychologists, and staff at CEBU. The average number of hours the psychologists spent on working with the families were reported as well.

#### Acceptability of Intervention and Study Procedures

Acceptability was measured with respect to: (a) the intervention, and (b) the study procedures. Participant’s dropout rate, session attendance, and duration of the intervention were registered.

Youths, parents, and teachers completed items related to treatment satisfaction at post- intervention. All items where rated on a 3-point scale: (0) “Not True,” (1) “Partly True,” and (2) “True.” For qualitative feedback about the program, open-ended questions were included to allow the participants to comment freely on what worked well and what needed to be improved in the B2S program.

At 12-month follow-up, youths and parents rated their satisfaction on the same 3-point scale and responded to open-ended questions about the family’s continuing use of strategies acquired in the B2S intervention.

### Measures Regarding Preliminary Outcome of the Intervention

The following measures were included as a part of the preliminary evaluation of B2S. The measures were planned to be outcomes in the RCT:

#### Primary Outcomes

##### School absenteeism

School absenteeism was measured using two different types of data. First, *school absenteeism (registry) data* were drawn from official school absenteeism records collected by the schools, provided by the municipality. The absenteeism score was calculated as a percentage of absenteeism in each of the following periods: (a) 4 weeks before the baseline questionnaires (baseline score); (b) 4 weeks after the post-intervention questionnaires (post score); (c) 2 weeks after the 3-month follow-up questionnaires (3-months follow-up score); and (d) 2 weeks after the 12-month follow-up questionnaires (12-months follow-up score).

Second, *school absenteeism (parent-report) data* was based on parent reports of the youth’s school-absenteeism at three occasions: (1) parents retrospectively reported the amount of school absenteeism the youths had the previous 3 months before inclusion in the study using the following categories: less than 10% (less than 6 schooldays), 10–20% (6–12 schooldays, which are about 1 day of absenteeism each week or biweekly), 20–30% (12–18 schooldays, which are about more than 1 day of absenteeism each week), 30–50% (18–30 schooldays, which are about 2–3 days of absenteeism each week), more than 50% (more than 30 schooldays which are 3 or more days of absenteeism each week), or 100% (the child has not attended school the last 3 months); (2) at the 3-month follow-up, parents retrospectively reported the youth’s school attendance for the 2 weeks prior to their completion of the questionnaires mailed to them, which was calculated to an absenteeism percentage score; and (3) the same applied at the 12-month follow-up.

#### Secondary Outcomes

##### Emotional, behavioral, and social difficulties

Youth emotional, behavioral and social difficulties was measured using the extended version of the *Strength and Difficulties Questionnaire* (SDQ; Goodman, 2001). The first part of the SDQ contains 25 items rated on a 3-point scale ranging from 0 to 2. Items are summed up into five subscales for emotional symptoms, conduct problems, hyperactivity/inattention, peer relationships problems, and prosocial behavior. The second part of the SDQ is an impact scale evaluating the level of chronicity, distress, social impairment, and burden to others of the problems reported. The scale contains five items (three items in the teacher version) rated on a 3-point scale ranging from 0 to 2. The SDQ includes both a child, parent, and teacher version. The Danish version of the SDQ has shown acceptable internal consistency (Cronbach’s α = 0.44–0.86) (Niclasen et al., 2012).

##### Anxiety

Youth anxiety was measured using the *Spence Children’s Anxiety Scale* (SCAS; Spence, 1998; Nauta et al., 2004). The scale contains 44 items (including six positive fillers in the child-version) rated on a 4-point scale ranging from 0 to 3. Items are summed up into six subscales for the specific anxiety diagnoses social phobia, panic disorder and agoraphobia, generalized anxiety disorder, obsessive–compulsive disorder, separation anxiety disorder, and fear of physical injury. The SCAS includes both a child (SCAS) and parent version (SCAS-P). The Danish versions of the SCAS and SCAS-P have demonstrated satisfactory test-retest reliability (SACS: *r* = 0.61–0.84, SACS-P: *r* = 0.53–0.88), and acceptable internal consistency (SCAS: Cronbach’s α = 0.59–0.92, SCAS-P: Cronbach’s α = 0.50–0.90 (Arendt et al., 2014).

##### Depression

Youth symptoms and levels of depression was measured using the *Mood and Feelings Questionnaire* (MFQ; Daviss et al., 2006). The MFQ includes both a child (33 items) and parent version (34 items), rated on a 3-point scale ranging from 0-2. Items are summed up into a total score. The Danish version of the MFQ has demonstrated high internal consistency (Cronbach’sα = 0.92–0.93) (Eg et al., 2018).

##### Self-efficacy

Youth self-efficacy was measured using the *Self-Efficacy Questionnaire for School Situations* (SEQ-SS; Heyne et al., 1998). The SEQ-SS contains 12 items about different situations associated with school attendance, each rated on a 5-point scale ranging from 1 to 5. The items are summed according to two subscales, Academic/Social Stress and Separation/Discipline Stress. A total score is calculated by summing all items (scores range from 12 to 60). Higher scores indicate a higher level of self-efficacy. The English version of the SEQ-SS has demonstrated high internal consistency (Cronbach’s α = 0.81–0.85) and good test–retest reliability (*r* = 0.79–0.91) (Heyne et al., 1998).

Parental self-efficacy was measured using the *Self-Efficacy Questionnaire for Responding to School Attendance Problems* (SEQ-RSAP; Heyne et al., 2016). The SEQ-RSAP contains 13 items concerning the parents’ level of self-efficacy in relation to helping their child attend school regularly and without difficulty. The items are rated on a 4-point scale ranging from 1 to 4. The items are summed to yield a total self-efficacy score (scores range from 13 to 52). Higher levels of reported self-efficacy are represented by a higher score. A preliminary unpublished study of a longer version demonstrated high internal consistency (Chronbach’s α = 0.91) and good test-retest reliability (*r* = 0.67) (Lavooi, 2010).

#### Additional Outcomes

The following measures were included as secondary outcomes in the RCT. Here they were included with the purpose of testing the feasibility of the length of all questionnaires in total:

##### Family functioning

Youths and parents reported on family functioning using the General Functioning subscale from *The McMaster Family Assessment Device* (FAD; Epstein et al., 1983).

##### Experience of being bullied

The *Personal Experience Checklist* (PECK; Hunt et al., 2012) is a questionnaire developed by Hunt et al. to provide a multidimensional assessment of a young person’s personal experience of being bullied.

##### Parent-school collaboration

Three items were developed to parents and teachers by the researchers to assess the quality of the collaboration between the parents and the school rated on a 4-point scale (from “not at all” to “very good”).

##### Pediatric quality of life

Youths reported their health-related quality of life using the *Child Health Utility 9D* index (CHU-9D; Stevens, 2012). The CHU-9D was developed for use in cost-utility analysis and therefore quality adjusted life years can be calculated (Canaway and Frew, 2013).

### Data Analysis

Descriptive statistics, including means, *SD*, and frequencies, were used to describe the sample characteristics, participant dropout rates, session attendance, intervention duration, and proportion of completed questionnaires.

Qualitative data based on the participants’ responses to the open-ended questions about the acceptability of the B2S program was collected and analyzed using a qualitative description design (Neergaard et al., 2009). The qualitative data were analyzed using content analysis with modifiable coding systems that corresponded to the data collected. The data was sorted to identify similar patterns and themes. Commonalities and differences among the data were also assessed. The codes were then grouped into six themes representing the general feedback from the participants about the intervention. The analyses were done by the first author and the coding were performed in NVivo (NVivo qualitative data analysis software; QSR International Pty Ltd. Version 12, 2018).

The preliminary evaluation of outcome included an evaluation of change over time on the outcome measures using Mixed Linear Models (MLMs). MLMs tolerate missing values and do not unnecessarily compromise statistical power. All MLMs were estimated with the *maximum likelihood method* (ML) and were based on the intent-to treat sample (*n* = 24). However, due to the small sample size, the *restricted estimate maximum likelihood method* (REML) is predicted to be the best fit, and was therefore used for the final model (Raudenbush and Bryk, 2002). The data were hierarchically arranged in two levels, with time at *Level 1* nested within individuals at *Level 2*. All models included a random intercept, and the slope was specified as random if improving the model fit evaluated by a significant change in the – 2LL fit statistics (Heck et al., 2013). Based on visual inspection of the data and an inspection of the model indices for the time variable on all outcome, the best fit for the time variable was evaluated for each model using – 2LL fit statistics (Heck et al., 2013). Covariance type was tested with Variance Components (VC), First-Order Autoregressive Structure [AR(1)], and Heterogeneous First-Order Autoregressive [ARH(1)], using the – 2LL fit statistics (Heck et al., 2013). The AR(1) or ARH(1) structure was used if it improved the model fit using – 2LL fit statistics (Heck et al., 2013).

Intervention effects were indicated by a significant change in means over time, indicated by a significant two-way interaction between participant’s scores and time. Effect sizes were expressed by Cohen’s *d*^1^, with 0.2, 0.5, and 0.8 considered as small, medium, and large effects respectively (Cohen, 1988). See Table 2, for an overview of the initial testing of the variables in the MLMs.

**TABLE 2:** Overview of the initial testing of the variables in the mixed linear models.

Outcome	Respondent	Method	Time	Covariance Type	Para.	Model
School Absenteeism (%)	Municipality	REML	TimeLog	VC	4	Random intercept and fixed slope

SCAS Total	Youth	REML	TimeLog	ARH(1)	6	Random intercept and random slope
	Parent	REML	Time	VC	4	Random intercept and fixed slope

SDQ – Emotional symptoms	Youth	REML	TimeLog	VC	4	Random intercept and fixed slope
	Parent	REML	TimeLog	VC	4	Random intercept and fixed slope
	Teacher	REML	TimeExp	VC	4	Random intercept and fixed slope

SDQ- Conduct problems	Youth	REML	Time	VC	4	Random intercept and fixed slope
	Parent	REML	TimeLog	VC	4	Random intercept and fixed slope
	Teacher	REML	TimeExp	VC	4	Random intercept and fixed slope

SDQ- Hyperactivity/inattention	Youth	REML	TimeLog	VC	4	Random intercept and fixed slope
	Parent	REML	Time2	VC	4	Random intercept and fixed slope
	Teacher	REML	TimeExp	VC	4	Random intercept and fixed slope

SDQ- Prosocial behavior	Youth	REML	TimeWeeks	VC	4	Random intercept and fixed slope
	Parent	REML	TimeLog	VC	4	Random intercept and fixed slope
	Teacher	REML	TimeWeeks	VC	4	Random intercept and fixed slope

SDQ- Problems with peers	Youth	REML	Time	VC	4	Random intercept and fixed slope
	Parent	REML	TimeLog	ARH(1)	6	Random intercept and random slope
	Teacher	REML	TimeExp	VC	4	Random intercept and fixed slope

SDQ Impact	Youth	REML	Time	VC	4	Random intercept and fixed slope
	Parent	REML	TimeLog	VC	5	Random intercept and random slope
	Teacher	REML	Time	VC	4	Random intercept and fixed slope

MFQ	Youth	REML	Time	VC	4	Random intercept and fixed slope
	Parent	REML	TimeLog	VC	5	Random intercept and random slope

SEQ-SS - Total	Youth	REML	Time2	ARH(1)	6	Random intercept and random slope
SEQ-SS -Academic	Youth	REML	Time	VC	4	Random intercept and fixed slope
SEQ-SS -Separation	Youth	REML	Time2	ARH(1)	6	Random intercept and random slope

SEQ-RSAP - Total	Parent	REML	TimeLog	ARH(1)	6	Random intercept and random slope

*REML, restricted Estimate Maximum Likelihood Method; TimeLog, log linear model of time; TimeExp, exponential model of time; TimeWeeks, modeling of time in weeks; Time2, quadratic model of time; ARH(1), first-Order autoregressive; VC, Variance Components.*

All statistical analyses were performed with IBM SPSS Statistics 25.00 for Windows (IBM Corp. Released 2016. IBM SPSS Statistics for Windows, Version 25.0. Armonk, NY, United States: IBM Corp).

## Results

### Recruitment Capability and Sample Characteristics

The sample consisted of 24 youths and their parents. Initial, the recruitment time were expected to take 1–2 months based on the eligible number of children in the municipality with more than ten percent absenteeism. However, it took 3 months to include the 24 youths.

As presented in Table 3, 24 youths aged 12.7 years (range 8–16 years) participated in the study. There was an equal number of girls and boys, and one fourth of the youths were totally absent from school across the last 4 weeks before study inclusion. For the majority of the youths the school had indicated to the parents that they were worried about the youths’ mental wellbeing. All youths had received treatment before study inclusion due to their absenteeism problems. Eight youths (33%) had one or more psychiatric diagnoses prior to inclusion, and they all had an anxiety disorder as one of their diagnoses. For the parents, 21% reported mental health problems themselves. In the semi-structured psychopathology interview, only one youth did not report any psychiatric symptoms. Symptoms related to anxiety and/or depression were most often reported (75% reported anxiety symptoms, 46% reported depressive symptoms).

**TABLE 3:** Sociodemographic characteristics of sample.

Characteristic	Participants
Age at inclusion, years, mean (*SD*)	12.7 (2.4)
Gender, males, *n* (%)	12 (50%)
Gender by age group, *n* (%)	
Males, aged 6–10 years	3 (25%)
Males, aged 11–16 years	6 (75%)
Females, aged 6–10 years	1 (8%)
Females, aged 11–16 years	11 (92%)
School absenteeism four weeks prior to inclusion, *n* (%)	
≤10% absenteeism 11–30% absenteeism	0 (0%) 4 (17%)
31–50% absenteeism	5 (21%)
51–70% absenteeism	5 (21%)
71–99% absenteeism	4 (17%)
100% absenteeism	6 (25%)
Academically behind peers (teacher-report), *n* (%)	8 (33%)
Educational support^1^, *n* (%)	5 (21%)
School/teacher worried about the youth’s mental wellbeing, *n* (%)	19 (79%)
Changed school at least once before inclusion, *n* (%)	8 (33%)
Changed school after inclusion, *n* (%)	10 (42%)
Former treatment due to absenteeism problems, *n* (%):	
School psychologist	16 (67%)
Private psychologist	13 (54%)
General practitioner	19 (79%)
Pediatric physician	4 (17%)
Child psychiatrics	16 (67%)
Other forms of help^2^	5 (21%)
No former treatment	0 (0%)
Current medication, *n* (%)	1 (4%)
Diagnosis prior to inclusion, *n* (%):	
Psychiatric diagnosis^3^	8 (33%)
Somatic diagnosis^4^	5 (21%)
Living with two parents, *n* (%)	11 (46%)
Maternal education (Intermediate or long), *n* (%)	16 (67%)
Paternal education (Intermediate or long), *n* (%)	8 (33%)
Ethnicity, *n* (%)	
Both parents born in DK	19 (79%)
One foreign born	5 (21%)
Two foreign born	0 (0%)
Maternal self-reported mental health problems, *n* (%)^5^	5 (21%)
Paternal self-reported mental health problems, *n* (%)^6^	4 (17%)
Symptoms reported in psychopathology interview, *n* (%)	
Anxiety symptoms	18 (75%)
Panic disorder	4 (17%)
Separation anxiety	6 (25%)
Social phobia	8 (33%)
Specific phobia	7 (29%)
Agoraphobia	7 (29%)
Generalized anxiety	5 (21%)
Obsessive Compulsive Disorder (OCD)	3 (13%)
Depressive symptoms	11 (46%)
Depressive symptoms – depressed mood/irritability	8 (33%)
Depressive symptoms – diminished interest or pleasure	10 (42%)
Depressive symptoms – fatigue or loss of energy	8 (33%)
Post-Traumatic Stress Disorder (PTSD)	2 (8%)
ADHD	4 (17%)
Oppositional defiant disorder	5 (21%)
Conduct disorder	1 (4%)
Pervasive or specific developmental disorders	6 (25%)
No symptoms reported	1(8%)
SRAS-R:	
Function 1: Avoidance of stimuli provoking negative affectivity, *n* (%)	17 (71%)
Function 2: Escape from aversive social and/or evaluative situations, *n* (%)	1 (4%)
Function 3: Pursuit of attention from others, *n* (%)	5 (21%)
Function 4: Pursuit of tangible reinforcement outside school, *n* (%)	0 (0%)
Function 1 and function 2 combined, *n* (%)^7^	1 (4%)

*^1^Number of youths receiving any educational support in the school (support teacher). ^2^Help from the social services in the municipality (*n* = 3), psychotherapist (*n* = 1), occupational therapist (*n* = 1). ^3^Anxiety(*n* = 8), autism (*n* = 4), learning difficulties (*n* = 2), depression (*n* = 1), OCD (*n* = 1), ADHD (*n* = 1), eating disorder (*n* = 1). ^4^Asthma or allergy (*n* = 4), constipation (*n* = 1). ^5^Anxiety(*n* = 5), depression (*n* = 4), ADHD (*n* = 2), autism (*n* = 1), learning difficulties (*n* = 1). ^6^Depression (*n* = 3), anxiety(*n* = 1), alcohol abuse (*n* = 1). ^7^Functional scores within 0.25 points of one another are considered equivalent.*

### Feasibility of Data Gathering Procedures

**FIGURE 1:**
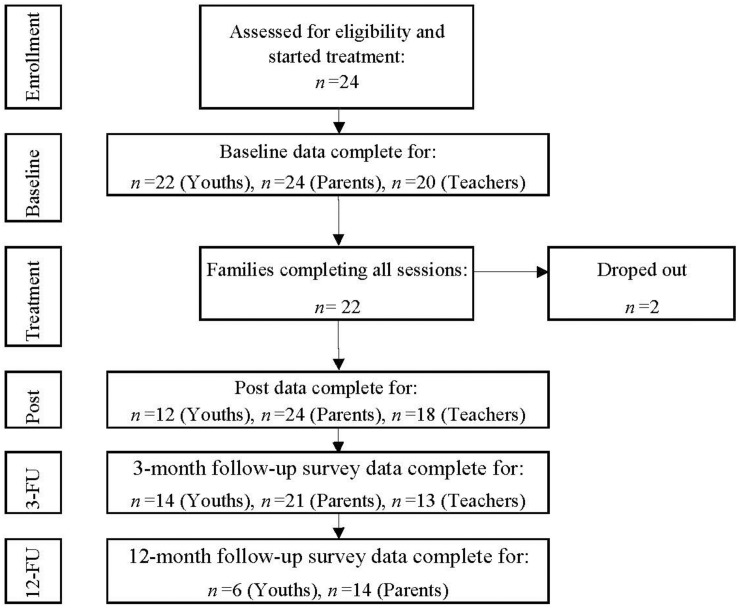
Flow diagram of response and completion rate.

As presented in Figure 1, in all cases, a parent completed the questionnaires at baseline and post-intervention, and in nearly all cases, a parent completed the questionnaires at 3-month follow-up (95%). However, the response rate declined at the 12-month follow-up, where almost two-thirds (64%) of the parents completed the questionnaires. The teachers’ completion rates were relatively high at baseline (83%) and post-intervention (86%). There was a decline in completion rates at 3-month follow-up (59%). When asked, teachers reported that they did not complete the questionnaires because they lacked sufficient knowledge regarding the youths in question because of their absenteeism from school. The response rates for the youths were high at baseline (92%), low at post-intervention (55%) and 3-month follow-up (64%), and very low at 12-month follow-up (27%).

The registry data was used in the analyses, as absenteeism was measured daily and not retrospectively and therefore viewed as the most accurate measure of school absenteeism. However, we replaced the registry data in the analyses with the parent-reported school absenteeism data in the following instances: (1) For seven of the participants (27%) their school absenteeism at baseline was reported as zero percentage in the registers, indicating that the schools did not register the absenteeism of the students. For these seven participants the parent-reported school absenteeism, at screening, were used instead of the registry data at baseline. (2) One participant (4%) was enrolled in a private school, therefore no registry data was available for this case, and the parent-reported school absenteeism was used instead. (3) After the summer break following the intervention, five youths (21%) changed to schools outside the municipality making registry data unavailable, thus parent-reported school absenteeism was used in these cases. (4) To investigate the robustness of the registry data, differences between the registry- and parent-reported data were compared for the three occasions where parent-reported data and registry data on school attendance were available (baseline, 3-month follow-up, and 12-month follow-up). A difference in the level of attendance was found at the 3-month follow-up for two cases (8%), where school absenteeism was significantly lower in the registry data compared to the parent-reported data (case 1: registry data = 10% and parent-reported data = 100%, case 2: registry data = 0% and parent-reported data = 70%). In these cases parent-report was used in the analyses.

### Resources to Implement the Intervention and Study Procedures

Based on evaluation with the psychologist two difficulties with the resources to manage the intervention was stated: Firstly, the psychologists spent more time on the cases than initially planned where we estimated an average of 30 psychology hours pr. case. This equals what the municipality estimates that psychologists spend on youth with SAP in their treatment as usual. In average however, the psychologists spent in average 40 h on each case. This included participation in sessions and school meetings, as well as preparation for the sessions and if necessary communication with the families between the sessions. Secondly, the psychologists reported feeling less competent in cases where youths’ primary problems were related to behavioral problems.

Based on evaluation of the resources to manage the study procedures with the staff and research team at CEBU there were enough resources to manage the technical part of the questionnaire collection. Office spaces, and administrative capacity were also evaluated as being sufficient.

### Acceptability of the Intervention

Of the 24 families who agreed to participate, 22 families (92%) completed the intervention. The two families (8%) who did not complete the intervention ended the intervention after session two and session six, respectively. The parents who withdrew after six sessions reported that their child found it too stressful to attend the sessions and that the setting with both parents, a psychologist, and a co-therapist attending the sessions made the child feel uncomfortable. The other family withdrew after two sessions because of lack of motivation to work with the child’s SAP as they were waiting for the child to attend a different school several months later.

With regards to participation, 19 of the 22 remaining families (86%) completed all 10 sessions, one family completed nine sessions, and two families completed eight sessions. The booster session was conducted with 19 families (86%). Thirteen (59%) of the cases included four school meetings as planned. One case did not include any school meetings. On average, the first school meeting was conducted 26 days after the first session (range 6–46 days). The mean duration of the B2S intervention (from the first session to the 10th session) was 80 days, with a range of 55–139 days. The intervention course was prolonged for three families, due to the summer holiday. On average, there were 76 days from the last session to the booster session with a range of 35–136 days. Again, due to the summer holiday the time between the last session and the booster was prolonged for most of the families. The whole B2S program, from assessment interview to booster session, spanned on average 182 days (range from 154 to 210 days).

#### Intervention Satisfaction

In general, both youth and parents were satisfied with B2S. As shown in Table 4, the majority of the youths and all parents answered ‘true’ or ‘partly true’ to the statement ‘If a friend needed similar help, I would recommend B2S,’ and all answered ‘true’ or ‘partly true’ to the statement ‘I trusted the therapist,’ All parents answered ‘true’ or ‘partly true’ to the statement ‘I have been given enough information about the purpose and course of B2S prior to the start,’ and all youths answered ‘true’ or ‘partly true’ to the statement ‘The therapist had an understanding of my worries and issues.’

**TABLE 4:** Intervention Satisfaction at post-intervention.

Item	Respondent	Response categories		
		*Not True*	*Partly True*	*Certainly True*
If a friend needed similar help, I would recommend Back2School	*Youth*	3 (25%)	3 (25%)	6 (50%)
	*Parent*	0 (0%)	6 (25%)	18 (75%)
	*Teacher*	2 (11%)	6 (33%)	10 (56%)

I trusted the therapist	*Youth*	0 (0%)	2 (17%)	10 (83%)
	*Parent*	0 (0%)	2 (8%)	22 (92%)
	*Teacher*	1 (6%)	7 (39%)	10 (56%)

I have been given enough information about the purpose and course of Back2School prior to the start	*Parent*	0 (0%)	3 (12%)	21 (88%)
	*Teacher*	2 (11%)	8 (44%)	8 (44%)

The therapist had an understanding of my worries and issues	*Youth*	0 (0%)	5 (42%)	7 (58%)

The meetings at the school was useful	*Teacher*	3 (17%)	9 (50%)	6 (33%)

*Data presented as *n* (%).*

Satisfaction as reported by the teachers was lower with regards to the statements ‘I trusted the therapist’ and ‘I have been given enough information about the purpose and course of B2S prior to the start.’ The majority of the teachers (83%) found the meetings at the school useful by reporting “partly true” or “true” to this statement.

At 12-month follow-up, all youths and 85% of the parents who completed the 12-month follow-up replied “partly true” or “”true” that they would still recommend B2S to a friend. Sixty-seven percent of the youth reported that they used the strategies from B2S, and 77% of the parents found the strategies helpful and a part of their everyday life. The B2S strategies which the parents still found helpful at 12-month follow-up were related to the specific cognitive behavioral techniques (e.g., graduated exposure, problem solving, rewarding, and cognitive restructuring).

#### Qualitative Feedback About the B2S Program

The participants’ responses to the open-ended questions about B2S were grouped within the six themes below. All participants completing the post-questionnaires (12 youths, 24 parents, and 18 teachers) responded to the open-ended questions and provided qualitative feedback.

##### Theme 1: assessment

Two parents and one teacher commented on the need for a better initial screening and assessment of the youth before the start of the program. One parent commented: *“It will be better for the children to be diagnosed before, to give a complete evaluation of what will be the most efficient help for the child.”* Another parent commented: *“I had hoped to find the answer to why my son was/is sad. He has indicated that there is ‘something’ that he found difficult to talk about that makes him sad. But we have never worked out what that is.”* Only one commented on the length of the questionnaires, where a parent reported that the questions were too difficult for an 8-year old.

##### Theme 2: the structure of the B2S program

Several parents commented on the structured and systematic approach of the B2S program, as a positive part of the program. The focus on both the youths’ strengths and difficulties was highlighted as well: *“It was very useful that both the child’s strengths and difficulties were identified.”* Parents viewed the inclusion of both the youths and their parents as a positive feature of the program. When asked about what worked well in the program, parents replied: *“That my daughter and I got a common language and techniques to work with her anxiety issues”* and “*That we were together in the program, the holistic perspective on the need of all family members to be aware of their behavior and thoughts.”* Others were positive about the inclusion of sessions with the parents only. One negative comment was reported regarding the inclusion of the parents in the intervention, where the parent stated that the presence of two therapists and parents could be too much for the youth compared to individual therapy only with the youth. Another parent mentioned that the therapist should be aware of adjusting the communication to a level understandable for the child and not just the parents. Two parents found it difficult to attend the sessions at the Center as their child found it difficult to get out of the house and therefore the child did not participate in the sessions.

##### Theme 3: the therapeutic techniques

Several participants commented on the usefulness of the graduated exposure. One youth commented: *“I have realized that to overcome my anxiety I have to face what triggers my anxiety.”* The rewards combined with the graduated exposure was also valued: “*It was really good and fun with the different types of rewards (stickers, praise) and the rewards that were given when doing graduated exposure.”* One youth recommended that the program in the future used more *in vivo* exposure. Several parents found the parent management techniques very helpful, including the implementation of new routines at home, techniques to manage conflict, and the support from the therapist making the parent’s more comfortable in making demands to their child.

##### Theme 4: collaboration with schools

Parents and teachers highlighted the importance of including the school in the intervention: *“The school makes an effort when there are meetings and especially follow-up meetings”* and *“As a school we got a better understanding of what anxiety is and how to plan a longer course for the child. As a teacher it can be difficult to know how to handle the situation or the student.”* The involvement of school management was also regarded as important: “*It is important that the school management is involved and is attending the meetings.”* Parents and teachers also commented on the timing of the school meetings, and suggested that the school meetings should be introduced earlier in the program: *“The school and B2S did not communicate in the beginning, which caused confusion because of contradictory guidance”* and *“It seems to be very useful to cooperate on helping the youth (family, school, B2S). However, we (the school) were involved too late in the program.”* Some of the teachers recommend that the therapist should gather more information about the student’s class and the social environment in the class: *“It is important that B2S focuses on what the child is a part of in the school. I would have liked it if the therapists came and observed the class and talked to the teacher, and thus got more information about what reality the child is coming back to.”* Some teachers also reported that there was a need for more information and clearer communication during the program: *”I needed more focus on how I, as a teacher, can handle different situations, to make sure that I am not working against what’s taught in B2S”* and *“Better communication, so everybody know what is expected from them.”*

##### Theme 5: timing, intensity, and duration of the program

Another theme from the participants’ feedback was the timing of the sessions. It was recommended by some of the parents to conduct the sessions before or after school hours. There was some disagreement in the comments regarding the intensity of the program. Some parents found the frequency of the sessions too intense and wanted more time between the sessions, while other highlighted the pace in the program as positive. Several parents commented on the duration of the B2S program, and suggested adding more sessions and an extra booster session after 1 year.

##### Theme 6: satisfaction with the therapists

All comments regarding the therapists from youth, parents, and teachers were positive, and reflected great satisfaction with the therapists: *“The therapists were very competent. It felt like they almost knew our son, even though they had only just met him. They were well-prepared,”* “*Very competent therapists, who knew how to make a good contact with our daughter without pressure. They were able to adhere to the manual without being too rigid,”* and “*The therapist gave me hope and motivation to do the things in the future, I want to.”*

#### Preliminary Outcome of the Intervention

**FIGURE 2:**
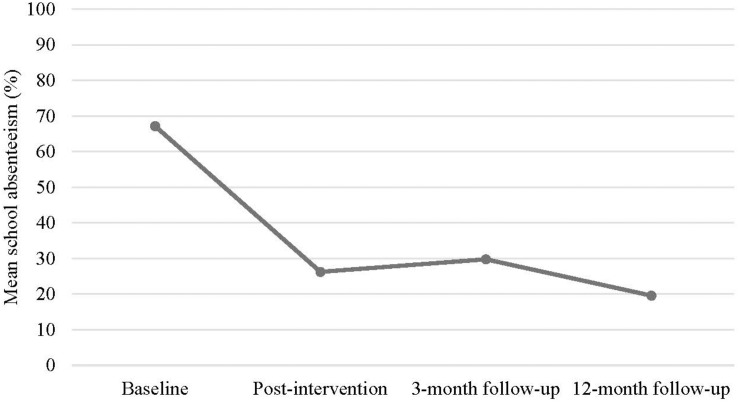
Mean school absenteeism from baseline to 12-month follow-up.

The level of school absenteeism was reduced on average from 67% at baseline to 26% at post-intervention and 20% at 12-month follow-up (see Figure 2). The change was significant (*p* = 0.001) with a large effect size (*d* = 1.357).

**FIGURE 3:**
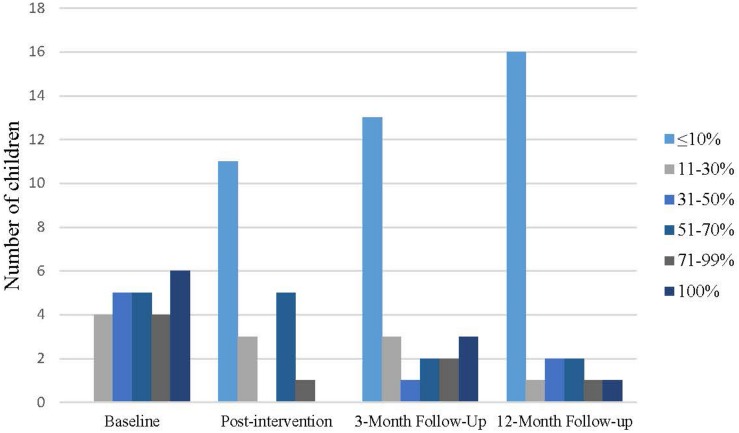
Level of school absenteeism.

As shown in Figure 3, at 12-month follow-up 16 (67%) of the participants were absent from school less than 10% of the time and therefore did not met the inclusion criteria with an absenteeism level of minimum 10% anymore. Four (17%) participants still attended school less than 50% of the time and one of the participant (4%) did not attend school at all at 12-month follow-up. At 3-month follow-up seven (29%) participant had more than 50% absenteeism and three (13%) were total absent from school.

As presented in Table 5, there was a significant average effect over time on several outcomes. All informants reported an average significant improvement on the SDQ emotional problem scale and the SDQ impact scale, all with large effect sizes. A significant and large effect on SDQ conduct problems was also found for parent- and youth report. No significant improvement was found on the SDQ hyperactivity scale, and a significant improvement was found only in youth-report on the SDQ peer problem scale, and prosocial behavior. For anxiety symptoms and depression symptoms, youth and parents reported on average a significant improvement with moderate to large effect sizes. On average, significant and large improvement in self-efficacy was also found for both youth and parent.

**TABLE 5:** Outcomes and estimates of intervention effects.

Outcome	Respondent	Baseline	Post-intervention	3-Month Follow-Up	12-Month Follow-Up	Time × Intervention effect
SDQ –	Youth	6.18 (2.34) [22]	4.33 (2.50) [12]	3.14 (2.25) [14]	2.83 (2.71) [6]	*F* = 37.303, *p* < 0.001, *d* = 2.040
Emotional	Parent	7.46 (2.02) [24]	5.29 (2.71) [24]	4.71 (2.57) [21]	3.71 (2.09) [14]	*F* = 45.01, *p* < 0.001, *d* = 1.744
symptoms	Teacher	6.20 (2.38) [20]	5.78 (2.24) [18]	4.77 (2.65) [13]		*F* = 4.449, *p* = 0.042, *d* = 0.709

SDQ- Conduct	Youth	1.82 (1.56) [22]	1.33 (0.98) [12]	0.86 (1.10) [14]	0.50 (0.84) [6]	*F* = 5.326, *p* = 0.028, *d* = 0.861
problems	Parent	2.04 (1.63) [24]	1.62 (1.38) [24]	1.24 (1.22) [21]	0.86 (0.95) [14]	*F* = 10.752, *p* = 0.002, *d* = 0.847
	Teacher	0.95 (0.89) [20]	1.62 (1.38) [24]	0.54 (0.78) [13]		*F* = 2.083, *p* = 0.157, *d* = 0.455

SDQ-	Youth	4.68 (2.34) [22]	3.33 (2.06) [12]	3.29 (1.54) [14]	3.33 (2.94) [6]	*F* = 3.708, *p* = 0.063, *d* = 0.661
Hyperactivity/	Parent	3.62 (2.55) [24]	3.92 (2.92) [24]	3.57 (2.38) [21]	3.57 (2.44) [14]	*F* = 0.079, *p* = 0.780, *d* = 0.072
inattention	Teacher	3.40 (2.28) [20]	3.92 (2.92) [24]	2.85 (2.48) [13]		*F* = 0.474, *p* = 0.495, *d* = 0.225

SDQ- Prosocial	Youth	7.32 (2.01) [22]	7.92 (2.07) [12]	7.93 (1.90) [14]	8.67 (1.21) [6]	*F* = 4.490, *p* = 0.041, *d* = 0.724
behavior	Parent	7.17 (2.06) [24]	7.42 (2.17) [24]	7.52 (2.11) [21]	7.57 (2.38) [14]	*F* = 2.25, *p* = 0.780, *d* = 0.072
	Teacher	6.40 (2.56) [20]	7.42 (2.17) [24]	7.77 (2.05) [13]		*F* = 4.144, *p* = 0.050, *d* = 0.696

SDQ- Problems	Youth	3.55 (2.09) [22]	2.92 (1.93) [12]	2.21 (1.93) [14]	1.50 (1.76) [6]	*F* = 8.484, *p* = 0.006, *d* = 0.958
with peers	Parent	2.63 (1.81) [24]	2.38 (1.64) [24]	2.00 (1.84) [21]	2.43 (2.28) [14]	*F* = 1.520, *p* = 0.229, *d* = 0.501
	Teacher	2.40 (2.11) [20]	2.38 (1.64) [24]	1.69 (1.60) [13]		*F* = 0.583, *p* = 0.451, *d* = 0.266

SDQ Impact	Youth	2.77 (2.71) [22]	1.75 (2.16) [12]	1.14 (2.21) [14]	1.17 (1.47) [6]	*F* = 6.974, *p* = 0.013, *d* = 0.918
	Parent	5.63 (2.16) [24]	3.63 (2.99) [24]	3.14 (2.80) [21]	2.93 (3.08) [14]	*F* = 15.701, *p* < 0.001, *d* = 1.488
	Teacher	3.95 (1.57) [20]	2.44 (2.73) [18]	1.08 (1.55) [13]		*F* = 31.427, *p* < 0.001, *d* = 1.915

SCAS Total	Youth	39.43 (16.77) [21]	32.50 (20.34) [12]	28.64 (17.18) [14]	24.84 (13.18) [6]	*F* = 5.101, *p* = 0.042, *d* = 1.256
	Parent	42.00 (16.18) [24]	34.95 (16.44) [22]	33.00 (16.88) [21]	28.21 (15.64) [14]	*F* = 22.385, *p* < 0.001, *d* = 3.229

MFQ	Youth	23.80 (12.13) [20]	17.33 (14.24) [12]	15.57 (13.19) [14]	11.33 (14.08) [6]	*F* = 4.954, *p* = 0.033, *d* = 0.763
	Parent	25.96 (10.00) [24]	18.91 (12.89) [22]	18.43 (13.79) [21]	16.46 (15.01) [13]	*F* = 6.531, *p* = 0.017, *d* = 1.002

SEQ-SS – Total	Youth	37.35 (12.14) [20]	41.83 (13.67) [12]	45.64 (11.75) [14]	51.17 (4.36) [6]	*F* = 4.824, *p* = 0.046, *d* = 1.206
SEQ-SS – Academic	Youth	18.25 (6.21) [20]	20.92 (6.64) [12]	22.36 (6.28) [14]	25.17 (2.64) [6]	*F* = 13.282, *p* = 0.001, *d* = 1.291
SEQ-SS – Separation	Youth	19.10 (6.66) [20]	20.92 (7.53) [12]	23.29 (6.09) [14]	26.00 (2.76) [6]	*F* = 4.649, *p* = 0.050, *d* = 1.171

SEQ-RSAP – Total	Parent	38.17 (4.19) [24]	41.96 (4.61) [22]	43.33 (6.37) [21]	44.23 (6.44) [13]	*F* = 11.489, *p* = 0.003, *d* = 1.489

*Data presented as mean (SD) [n].*

## Discussion

This study of the acceptability of the B2S intervention and the feasibility of evaluating it an RCT study informs a range of modifications to be made. Following, we discuss modifications to recruitment, data gathering, and resourcing. Thereafter, we discuss the acceptability and preliminary effectiveness of B2S.

### Recruitment and Sample Characteristics

Twenty-four youth and their parents were recruited, although it took more time to recruit the targeted number of families than was anticipated. This could be due to the fact that it was difficult to disseminate information about the intervention to parents in the municipality. Not all schools used their information channels to inform parents about the intervention. It was also difficult to get information about the B2S program to relevant professionals (e.g., social workers, psychologists). Because families self-refer to the B2S program, it is important that information about the intervention reaches families in need. Thus, for the RCT, the municipality will make it mandatory for all schools to inform parents about B2S. Before starting the RCT, more effort would be made to get information to relevant professionals, including sending information about B2S to teachers at all schools within the municipality.

The inclusion criterion of 10 percent absenteeism during the last 3 months might be regarded by some as a low threshold for inclusion. However, by using this lower threshold, the results would seem to be relevant to the broader population of youth with SAPs and not only to the smaller group of youth with severe SAPs (e.g., complete absenteeism for the last 6 months). Despite our low threshold for inclusion, most youth who were included in the feasibility study had high levels of school absenteeism, and high scores on measures of anxiety and depression. Only one youth reported no symptoms during the psychopathology interview. In short, while the inclusion criteria permitted referral of youth with mild SAPs, the families of youth with more severe problems sought help via the B2S program.

### Data Gathering Procedures and Outcome Measures

The percentage of parents who responded to the questionnaires at baseline, post-intervention and 3-months follow-up was acceptable, except at the 12-month follow-up. In cases where either parents or youth did not complete the questionnaires within 2 weeks, a reminder email was sent on two occasions. Nevertheless, the response rate among youths was low, both after the intervention and at follow-up. None of the youth and just one parent commented on the length of the questionnaires (that it was too long), suggesting that the low response rate among youth was not due to the extensive number of items in the questionnaires. Some of the youths refused to complete the questionnaires or the parents exempted their child from completing the questionnaires, believing that is was too challenging for them. Thus, in the RCT, the importance of completing the questionnaires would be highlighted for the psychologists, co-therapists, as well as the parents and youth. It would be mandatory for the youth and parents to complete the baseline measures to be included in the RCT. In the RCT, in addition to the email reminders, participants not completing the questionnaires would receive a telephone reminder. Because we expect a lower response rate in the control group, participants in the control group would receive a shorter version of the post-intervention assessment battery, and families would be offered a gift card (value 200 DKK/26 EUR) after the completion of post-intervention assessment and again after follow-up.

At 3-month follow-up the response rate among the teachers was low, largely attributable to the fact that 10 youth changed school after the completion of the intervention. The 3-month follow-up questionnaires was collected shortly after the youth’s change of school, and therefore the teachers at the new school thought that they did not know the students well enough to complete the questionnaires.

The absenteeism data from the school register was intended to be our primary outcome measure. However, a comparison of parent-reported absenteeism and absenteeism based on school register data suggests that the validity of the school-registered absenteeism was questionable for some youths. In the RCT, we would therefore include a detailed parent registration of the youths’ daily attendance during the last 2-weeks before each data-collection points (pre-intervention, post-intervention, and follow-up), to be able to check this registration against the school’s registration.

### Resources and Ability to Implement the Study and Intervention

There were two main difficulties with respect to resourcing and ability to deliver the intervention. First, the psychologists spent more time than initially planned on the preparation of sessions, but we expect that the time used per case would be lower in the RCT because the psychologists would be more familiar with study procedures and the intervention itself. However, as a precaution against potential overburdening of the psychologists, two additional psychologists from the municipality would be trained for participation in the RCT. Furthermore, in the RCT, measures of implementation cost and health related benefits will be collected for both the B2S group and treatment as usual group to conduct cost-benefit and cost-utility analyses of the B2S program.

Second, the psychologists were school psychologist with counseling as their main task before participating in B2S. The psychologists received a 6-day training course and weekly face-to-face group case supervision. Based on the preliminary results the competences of the psychologists to use B2S seems sufficient. However, because the psychologists reported feeling less competent in cases where youths’ primary problems were related to behavioral problems, a supervisor with expert knowledge about externalizing problems and parent management techniques would be included as a supervisor in the RCT. Other matters related to resourcing were not found to be problematic (e.g., setting up the digital questionnaires and monitoring the questionnaires collections, office space, and administrative capacity).

### Acceptability of the Study Procedures and Intervention

The dropout rate of 8 percent is comparable to or lower than other studies examining the effect of therapy for school refusal (Heyne and Sauter, 2013). Moreover, 86 percent of the families participated in all intervention sessions. In general, parents and youth were satisfied with B2S, and satisfaction was maintained 1 year after the intervention. At the 1-year follow-up, the majority of families reported that they had implemented the strategies they acquired during the B2S sessions. The teachers’ satisfaction ratings were lower than those of parents and youth, but the majority of the teachers found the meetings at the school useful.

Parent qualitative feedback indicated that some parents wished there had been a more comprehensive diagnostic screening of the youth before the start of the intervention. These were the families for whom symptoms of more complex mental health problems were identified among the youth during their participation in B2S. The B2S psychologists referred these families to psychiatric specialists for a diagnostic screening of the youth. Because the initial screening in B2S already comprised a comprehensive battery of questionnaires, together with the assessment interview, this procedure will not be changed in the RCT.

The family oriented approach was highlighted by the parents in the qualitative feedback as positive, and the parents found the parent management techniques very useful. In addition, the involvement of the school was mentioned as an important part of the B2S program by parents and teachers. Based on the qualitative feedback from teachers and parents, when B2S is implemented in the context of an RCT the school meetings would be scheduled earlier in the program, and a detailed agenda for the meetings would be included in the B2S manual. Two of the parents would have preferred that the sessions were conducted in the home rather that at the clinic because the child did not wanted to leave the house. In these cases the intervention was focused on the parents’ behavior, and the parents were taught strategies to work with the child at home. They would be guided in how to help their child attend therapy sessions at the Center, constituting graded exposure for the child with respect to leaving the house, as a step toward ultimately being able to attend school.

### Preliminary Outcome of the Intervention

One of the inclusion criteria for participating in the study was absenteeism above 10 percent. Following the B2S program, the number of youths with levels of school absenteeism below 10 percent were increasing from 45 percent of the youth at post-intervention to 54 percent at 3-month follow-up and 66 percent 1 year after the intervention. The large reduction in school absenteeism was comparable to or better than two previous non-controlled studies with youth with SAPs (Heyne et al., 2011; Hannan et al., 2019). However, the youth in those studies were older and presented with more psychological symptoms, perhaps explaining the larger improvement in school attendance in our sample.

B2S includes modules targeting anxiety, depression, and behavioral problems. We observed significant and large reductions over time with respect to each of these areas of youth functioning. This highlights the relevance of these modules in the intervention as it seems that the intervention do address these problems in the youth. Due to the uncontrolled design, the improvement seen in the outcome measures cannot for sure be related to B2S. However, based on this study the inclusion of both the intervention elements as well as outcomes seems relevant for the upcoming RCT.

In addition, the youth and their parents reported a higher level of school-related self-efficacy after the intervention. Specifically, youth felt more able to cope with challenging school situations and parents were more confident about responding to their child’s SAP. Because of the change in self-efficacy, and preliminary support for the role of increased self-efficacy in mediating outcomes following treatment for school refusal (Maric et al., 2013), the RCT would include self-efficacy as a mediator variable, measured at two time points during the intervention. This would provide greater insight into the impact of self-efficacy on school attendance and vice versa.

### Limitations

There are a number of limitations to the current study. First, the design was uncontrolled and therefore the impact of B2S on the positive changes observed on the outcome measures is not clear. The positive changes may be related to other factors such as spontaneous remission or regression toward the mean. Second, because of the uncontrolled design of the study, the acceptability of randomization and its impact on attrition could not be evaluated. Third, the proportion of youth completing the questionnaires was low. This was especially the case for the 12-months follow-up were only 27 percent of the youth completed the questionnaires. Third, the validity of absenteeism data from the school register was questionable for some of the youths as the schools had registered 27 percent of the youth as having no school absenteeism at baseline.

## Conclusion

In conclusion, this study of the feasibility of the B2S program found high participation rates as well as high levels of satisfaction with the program which were maintained 1 year after the intervention. Teacher satisfaction was lower than that of youth and parents, but the majority found the school’s participation in the intervention helpful. Preliminary evaluation of intervention outcomes showed a significant increase in school attendance and decrease in psychological symptoms, as well as a significant increase in self-efficacy for both youth and parents.

The study signaled areas for improvement. The main adaptation made to the B2S manual was to increase emphasis on the importance of the school meetings and the timing of these. Several adaptations to the study procedure were also identified. First, to ensure adequate recruitment for the RCT more effort will be made to get information about the B2S program to professionals in the municipality and to parents. Second, parent-reported school absenteeism data will be collected at all time-points to test the validity of the register-based school absenteeism data. Finally, more psychologist resources are needed because it was more time-consuming for the psychologists to implement B2S than expected. Accounting for these adaptations it seems feasible to evaluate the effectiveness of B2S in a RCT.

## Data Availability Statement

The datasets generated for this study are available on request to the corresponding author.

## Ethics Statement

Ethical review and approval was not required for the study on human participants in accordance with the local legislation and institutional requirements. Written informed consent to participate in this study was provided by the participant, and the participants’ legal guardian/next of kin where appropriate.

## Author Contributions

JL is the corresponding author and drafted the manuscript. MT is the principal investigator. MT and JL obtained funding for the project. MT, DJ, and JL designed the study. WS, PJ, and DH are members of the advisory board for the project. WS and DH advised in the design of the study. PJ developed the psychopathological interview used in the study. All authors were involved in the writing and editing of the manuscript.

## Conflict of Interest

The authors declare that the research was conducted in the absence of any commercial or financial relationships that could be construed as a potential conflict of interest.

## Footnotes

1 Effect-size equation (Cohen’s *d*): *d* = 2 × √(F/*df*)
